# Perceived Income Adequacy Versus Household Income as a Measure of Socioeconomic Status in 6 Countries, 2022-2023 International Food Policy Study

**DOI:** 10.1177/00333549251358655

**Published:** 2025-08-20

**Authors:** Rachel B. Acton, Christine M. White, Vicki L. Rynard, David Hammond

**Affiliations:** 1 School of Public Health Sciences, University of Waterloo, Waterloo, ON, Canada

**Keywords:** income, surveys and questionnaires, socioeconomic factors, food security

## Abstract

**Objectives:**

Household income is a common indicator of socioeconomic status in population surveys; however, measures such as perceived income adequacy are increasingly used as alternatives. We used a multicountry dataset to explore the utility of perceived income adequacy as compared with household income, focusing on missing data rates, associations with household food security, and responses from young people versus parents.

**Materials and Methods:**

We conducted online surveys in 2022-2023 among adults (n = 50 913) and young people (aged 10-17 y; n = 23 013) as part of the International Food Policy Study in Australia, Canada, Chile, Mexico, the United Kingdom, and the United States. We used descriptive analyses to examine missing data for income adequacy and household income adjusted for household size. We used linear regression models to test the association between the income measures, their associations with household food security, and their correspondence in reported income adequacy between young people and parents.

**Results:**

The proportion of missing data was greater for household income (5.3%; n = 2688) than for income adequacy (1.0%; n = 488). Income adequacy and household income were positively correlated (*r* = 0.25-0.44; *P* < .001 for all countries). Both measures independently predicted household food security (*P* < .001 for all countries), with a stronger association observed for income adequacy. Family income adequacy reported by young people was strongly associated with parental reports (*r* = 0.47-0.62; *P* < .001 for all).

**Practice Implications:**

Perceived income adequacy may be preferrable to traditional household income measures for assessing the effect of financial position on health-related outcomes, particularly among young people and older or retired populations, for whom household income may be difficult to report.

Financial position is a key indicator of socioeconomic status and is strongly associated with a range of health outcomes.^1-4^ In population-level surveys, financial position has traditionally been assessed by measures of total household income. For example, most national surveillance surveys ask respondents to report their household’s total annual or monthly income from all sources.^5^ Household income is useful for quantifying respondents’ income relative to other respondents or income cutoffs such as the poverty line, and it is associated with outcomes including self-rated health, chronic disease, and mental health.^5-7^ However, using household income in population surveys has several limitations. Household income does not capture factors that may affect an individual’s financial well-being, such as spending patterns, expectations, debt, or access to other financial resources.^5^ Income measures are also prone to missing data due to respondents being unwilling to respond, uncertain how to quantify household income, or simply not knowing their household income (eg, in the case of minors and dependents).^8-10^ Furthermore, household income has been shown to have weaker associations than subjective financial well-being with health outcomes among subgroups that are less reliant on traditional income sources, such as young adults and older adults.^6^

Alternative measures may avoid some of the limitations of household income, such as subjective social status,^11,12^ financial hardship,^13^ or perceived income adequacy (also referred to as subjective financial well-being or financial strain).^5-7,14^ Each measure aims to estimate the social or financial position of a household or individual by asking respondents to quantify concepts such as their relative social standing, difficulty in making monthly payments, or satisfaction with their present financial situation. Of these, the current study focused on perceived income adequacy. Several studies have explored the viability of perceived income adequacy as a measure to estimate indicators of health.^5,14^ Perceived income adequacy captures subjective perceptions of financial well-being or ability to make ends meet.^5^ Research has suggested that perceived income adequacy may be a strong predictor of health outcomes, particularly among older groups.^5-7,14,15^ However, most studies are limited in size and/or to a specialized population (eg, older adults). To our knowledge, no studies have explored the suitability of measures of perceived income adequacy among young people. Additional data on the utility of perceived income adequacy as compared with household income measures have the potential to inform researchers and practitioners exploring the relationship between financial position and health.

Thus, the current study explored perceived income adequacy as a measure of socioeconomic status in a large, multicountry, repeat cross-sectional survey dataset among adults and young people. Our study had 4 primary objectives: (1) to examine differences in missing data (between income adequacy and an established measure of household income adjusted for household size); (2) to examine associations between perceived income adequacy and adjusted household income; (3) to compare the strength of association of perceived income adequacy and adjusted household income with household food insecurity, a common indicator used to assess the health-related effects of financial status; and (4) to examine correspondence between perceived income adequacy reported by young people and their parents or guardians.

## Materials and Methods

### Data Sources

Data were from the 2022 and 2023 waves of the International Food Policy Study Adult and Youth surveys, annual repeat cross-sectional surveys conducted in Australia, Canada, Mexico, the United Kingdom, the United States, and Chile (youth surveys only).^16^ We collected data via self-completed web-based surveys conducted in November–December 2022 and 2023, separately with adults aged 18 to 100 years and young people aged 10 to 17 years.

Respondents for the adult surveys were recruited through Nielsen Consumer Insights Global Panel, Qualtrics, and their partners’ panels. Adult respondents provided consent prior to survey completion and received remuneration in accordance with their panel’s usual incentive structure (eg, points-based or monetary rewards).

We recruited respondents for the youth surveys through parents or guardians enrolled in the Nielsen Consumer Insights Global Panel and their partners’ panels. The survey panels sent invitations with unique survey links to adult panelists in each country. For panelists who confirmed that they had children or adolescents aged 10 to 17 years living in their households, we asked for permission to invite them to complete the survey, although only 1 child or adolescent per household was invited. We then provided the children and adolescents with information about the study and asked for their assent.

We conducted the surveys in English in Australia and the United Kingdom, English or French in Canada, Spanish in Chile and Mexico, and English or Spanish in the United States. Professional translation services translated the French and Spanish surveys, with additional review by members of the research team who were native in each language. The analytic sample included a total of 50 913 adults (26 273 in 2022 and 24 640 in 2023) and 23 013 young people (11 492 in 2022 and 11 521 in 2023).

The University of Waterloo Research Ethics Board (REB 30829 and 41477) reviewed and approved this study. A full description of the study methods can be found in the International Food Policy Study Adult and Youth technical reports.^17^

### Measures

We adapted a measure of perceived income adequacy from previous studies^6,15^ with minor wording changes. In the adult surveys, the measure asked, “Thinking about your total monthly income, how difficult or easy is it for you to make ends meet?” The response options were as follows: very difficult, difficult, neither easy nor difficult, easy, very easy, don’t know, and refuse to answer. In the youth surveys, the parent or guardian of each responding young person was first asked the aforementioned measure of income adequacy before the child or adolescent completed the survey. The survey later assessed perceived income adequacy among young respondents by asking, “Does your family have enough money to pay for things your family needs?” Response options were as follows: not enough money, barely enough money, enough money, more than enough money, don’t know, and refuse to answer. Aside from direct translation, we deemed no wording adjustments necessary to adapt these measures to the local dialect of each country. Previous research has validated the adult measure of perceived income adequacy for use among older European adults.^15^ No studies have tested the validity and reliability of the youth measure in its current form, but we designed the measure to be appropriate for the literacy levels of people aged as young as 10 years, and the measure performed well in cognitive testing.

We assessed adjusted household income in the adult surveys by asking respondents to select their total household income from a range of 11 to 13 response options, depending on the country.^17^ We then adjusted this household income for self-reported household size (Supplemental Material) and categorized the resulting values into quintiles for comparisons across countries.

We assessed household food security in the adult surveys using the 10-item (for households without children) or 18-item (for households with children) Household Food Security Survey Module, which queries respondents about a range of food experiences (eg, worrying about running out of food, cutting the size of meals, skipping meals).^18,19^ We then summed the number of affirmative responses and rescaled them to produce values ranging from 0 to 9.3 to allow for comparisons between households with and without children, per the Household Food Security Survey Module guidelines.^20^ Higher scores indicated a higher likelihood of household food insecurity. For descriptive analyses, we further categorized respondents into food secure (score, 0 to <2.4), low food security (score, 2.4 to <4.7), or very low food security (score, 4.7-9.3).^20^

Other sociodemographic measures included age, sex, highest level of education (adults only), ethnicity, and employment status.

### Descriptive Analyses

We used descriptive analyses to present the frequency and distribution of sociodemographic characteristics, missing data, and the variables of interest.

#### Perceived income adequacy and household income

We used bivariate linear regression models to calculate Pearson correlation coefficients between adults’ income adequacy and adjusted household income quintiles. Even though the measures used here are ordinal, the underlying construct is continuous, and parametric methods are known to be robust to departures from assumptions, particularly when sample sizes are large.^21^ We stratified models by country.

#### Perceived income adequacy and adjusted household income as predictors of food security

We used bivariate linear regression models to test the associations for (1) income adequacy and (2) adjusted household income quintiles with the household food security scale (0-9.3). We stratified models by country. In a subsequent step, we added age, ethnicity, education, and employment status and their 2-way interactions with the income variables separately to the models to test differences in the associations across levels of each variable.

#### Perceived income adequacy among young people versus parents or guardians

We used bivariate linear regression models to calculate the Pearson correlation coefficients of income adequacy between young people and their parents or guardians. We stratified models by country.

For all models, we omitted respondents with missing data (don’t know or refuse) for the outcomes and covariates of interest. We weighted data with poststratification sample weights constructed by a raking algorithm with population estimates from the census in each country.^17^ We weighted all reported estimates. We interpreted Pearson correlation coefficients according to the guidelines outlined previously,^22,23^ with values ranging from ±0.65 to ±1.0 suggesting a strong correlation, ±0.35 to <±0.65 indicating a moderate correlation, and <±0.35 indicating a weak correlation. We used SAS Enterprise Guide version 8.4 (SAS Institute Inc) to conduct analyses.

## Results

### Sample Characteristics and Missing Data

Adult respondents were approximately evenly distributed across age, sex, and education groups, with the exceptions of Mexico and the United States, where most respondents had low levels of education (eTable 1 and eTable 2 in Supplemental Material). Most adult respondents were categorized as majority ethnicity, tended to report income adequacy ranging from difficult to easy, and lived in food-secure households. About half of respondents in each country reported their main activity in the past week to be paid work. Children and adolescent respondents were approximately evenly distributed across age and sex groups. Most were categorized as majority ethnicity and stated that their family had enough money.

We found a greater proportion of missing responses for household income (don’t know, refuse, or missing household size; 5.3% across all countries) as compared with income adequacy (1.0%; eTable 3 in Supplemental Material). Adults aged 18 to 29 years had the highest proportion of missing data among age groups for both measures but to a greater extent for household income (7.1%) than for income adequacy (2.4%).

### Perceived Income Adequacy and Household Income

**Figure 1.**
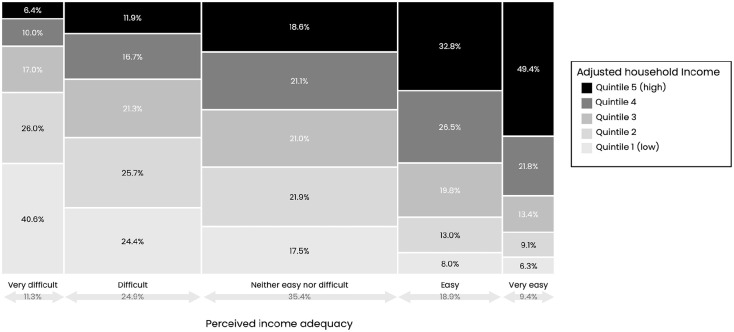
Distribution of adjusted household income quintiles by perceived income adequacy among adult respondents in the 2022-2023 International Food Policy Study surveys across 5 countries (Australia, Canada, Mexico, United Kingdom, United States), weighted by survey weight.^17^ Perceived income adequacy was measured by the following question: “Thinking about your total monthly income, how difficult or easy is it for you to make ends meet?” Household income was adjusted for self-reported household size and categorized into quintiles, with quintile 1 representing respondents with the lowest adjusted household income and quintile 5 representing respondents with the highest adjusted household income.

Most respondents who reported that it was very difficult to make ends meet had household incomes in the lowest 2 quintiles, while most respondents reporting that it was easy or very easy to make ends meet had household incomes in the highest 2 quintiles (Figure 1).

Pearson correlation coefficients indicated weak to moderate positive correlations between income adequacy and adjusted household income in Australia (*r* = 0.33), Canada (*r* = 0.43), Mexico (*r* = 0.25), the United Kingdom (*r* = 0.39), and the United States (*r* = 0.44; all *P* < .001).

#### Perceived income adequacy and adjusted household income as predictors of food security

**Figure 2.**
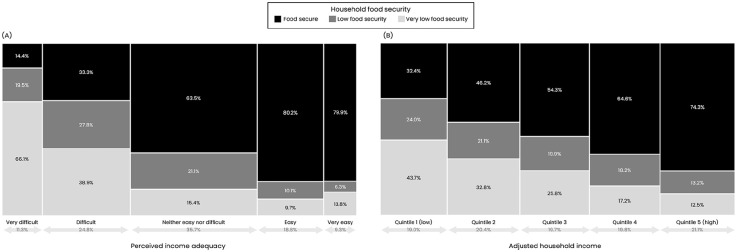
Distribution of household food security across levels of (A) perceived income adequacy and (B) adjusted household income among adult respondents in the 2022-2023 International Food Policy Study surveys across 5 countries (Australia, Canada, Mexico, United Kingdom, United States), weighted by survey weight.^17^ Perceived income adequacy was measured by the following question: “Thinking about your total monthly income, how difficult or easy is it for you to make ends meet?”

Two-thirds (66.1%) of adults who reported very difficult income adequacy had responses indicating very low food security, as compared with 43.7% of adults in the lowest household income quintile (Figure 2). *R*^2^ and *F* values were higher for income adequacy (*R*^2^ = 0.164-0.318; *F* = 272.3-556.2) than for household income (*R*^2^ = 0.056-0.147; *F* = 88.4-233.8) in each country (Table 1), indicating that income adequacy explained a greater proportion of the variance in food security than household income.

**Table 1.** *R*^2^ and Wald *F* test values for bivariate linear regression models testing the associations of perceived income adequacy and adjusted household income with household food security scale among adult respondents in the 2022-2023 International Food Policy Study surveys, stratified by country, weighted by survey weight^a^

	Perceived income adequacy^b^ as a predictor of household food security^c,d^		Adjusted household income^e^ as a predictor of household food security^c,f^	
Country	*R* ^2^	Overall model *F*^g^	*R* ^2^	Overall model *F*^g^
Australia (n = 7695)	0.241	424.1	0.056	88.2
Canada (n = 8317)	0.322	564.3	0.147	195.6
Mexico (n = 10 849)	0.162	263.7	0.142	236.1
United Kingdom (n = 7562)	0.247	445.4	0.086	128.2
United States (n = 13 487)	0.192	466.0	0.088	181.9

a Data source: International Food Policy Study 2022 and 2023 Adult surveys.^17^

b Perceived income adequacy was assessed by asking, “Thinking about your total monthly income, how difficult or easy is it for you to make ends meet?” Response options were on a 5-point Likert scale: 1 = very difficult, 2 = difficult, 3 = neither easy nor difficult, 4 = easy, and 5 = very easy. Participants responding don’t know or refuse were omitted from this analysis.

c Household food security was assessed by using the 18-item Household Food Security Survey Module, rescaled to scores ranging from 0 to 9.3.

d Linear regression models with household food security scale as the dependent variable and perceived income adequacy as the independent variable.

e Adjusted household income was assessed by asking respondents to select their total household income from a range of 11 to 13 response options, depending on the country. Reported household income was then adjusted for self-reported household size and categorized into quintiles. Participants responding don’t know or refuse were omitted from this analysis.

f Linear regression models with household food security scale as the dependent variable and adjusted household income (quintiles) as the independent variable.

g Each value in column is significant at *P* < .001.

In a subsequent step, we observed 2-way interactions between each income measure and age, ethnicity, education level, and employment status (*P* < .001 for all). Therefore, we stratified models by age, ethnicity, education, and employment groups. The *R*^2^ and *F* values remained higher for income adequacy than for household income in all groups (Table 2). Income adequacy accounted for the least amount of variance in food security among adults aged 18 to 29 years and the most amount of variance among adults aged ≥60 years. In contrast, household income explained the second-least amount of variance among respondents aged ≥60 years. Both income measures explained the least amount of variance in food security among respondents reporting a minority ethnicity and those with high education levels. Apart from the other employment category, income adequacy explained the most amount of variance in household food security among retired respondents, while household income explained the most amount of variance for those reporting paid work, parental leave, or long-term disability in the past week.

**Table 2.** *R*^2^ and Wald *F* test values for bivariate linear regression models testing the associations of perceived income adequacy and adjusted household income with household food security among adult respondents in the 2022-2023 International Food Policy Study surveys, across 5 countries,^a^ stratified by age and education groups, weighted by survey weight^b^

	Perceived income adequacy^c^ as a predictor of household food security^d,e^		Adjusted household income^f^ as a predictor of household food security^d,g^	
Characteristic	*R* ^2^	Overall model *F*^h^	*R* ^2^	Overall model *F*^h^
Age, y				
18-29	0.128	233.4	0.068	119.1
30-44	0.174	459.5	0.089	195.3
45-59	0.307	833.6	0.118	262.9
≥60	0.371	626.4	0.070	105.5
Ethnicity^i^				
Majority	0.273	1931.6	0.092	531.8
Minority	0.154	337.7	0.085	178.8
Education level^j^				
Low	0.244	949.3	0.083	274.7
Medium	0.275	748.5	0.081	187.2
High	0.130	418.2	0.041	118.7
Employment status^k^				
Paid work	0.184	890.5	0.096	411.5
Unemployed or unpaid work	0.186	390.1	0.077	149.8
Retired	0.375	429.9	0.089	100.9
Parental leave or long-term illness/disability	0.329	130.4	0.096	31.8
Other	0.392	50.4	0.108	6.6

a Australia, Canada, Mexico, the United Kingdom, and the United States.

b Data source: International Food Policy Study 2022 and 2023 Adult surveys.^17^

c Perceived income adequacy was assessed by asking, “Thinking about your total monthly income, how difficult or easy is it for you to make ends meet?” Response options were on a 5-point Likert scale: 1 = very difficult, 2 = difficult, 3 = neither easy nor difficult, 4 = easy, and 5 = very easy.

d Household food security was assessed by using the 18-item Household Food Security Survey Module, rescaled to scores ranging from 0 to 9.3.

e Bivariate linear regression models with household food security scale as the dependent variable and perceived income adequacy as the independent variable.

f Adjusted household income was assessed by asking respondents to select their total household income from a range of 11 to 13 response options, depending on the country. Reported household income was then adjusted for self-reported household size and categorized into quintiles.

g Bivariate linear regression models with household food security scale as the dependent variable and adjusted household income (quintiles) as the independent variable.

h Each value in column is significant at *P* < .001.

i Ethnicity was assessed by using country-specific race and ethnicity categories and analyzed as a binary variable (majority/minority) to accommodate comparisons across countries. Ethnicity categories were recoded as follows: (1) Australia majority = only speaks English at home, minority = speaks a language other than English at home or indicated they are aboriginal or Torres Straight Islander; (2) Canada majority = White, minority = other ethnicity; (3) Mexico majority = non-Indigenous, minority = Indigenous; (4) United Kingdom majority = White, minority = other ethnicity; (5) United States majority = White, minority = other ethnicity.

j Participants were asked, “What is the highest level of formal education that you have completed?” Responses were categorized as low (completed secondary school or less), medium (some postsecondary qualifications), or high (university degree or higher) according to country-specific criteria.

k Participants were asked, “What was your main activity in the past week?” Responses were categorized into paid work (working at a paid job or business; vacation [from paid work]), unemployed or unpaid work (looking for paid work; going to school [including vacation from school]; caring for children; household work; volunteering; caregiving other than for children); retired (retired), parental leave or long-term disability (maternity/paternity leave; long-term illness [or disability]), or other (other open-text responses [eg, short-term illness]).

#### Perceived income adequacy between young people and parents or guardians

**Figure 3.**
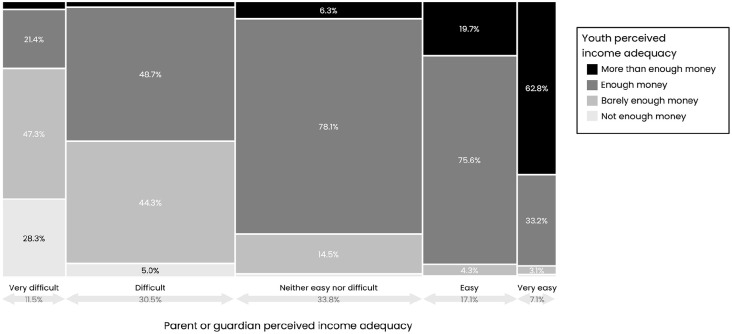
Distribution of children’s and adolescents’ perceived income adequacy by their parents’ or guardians’ perceived income adequacy among respondents to the 2022-2023 International Food Policy Study Youth surveys across 6 countries (Australia, Canada, Chile, Mexico, the United Kingdom, the United States), weighted by survey weight.^17^ Perceived income adequacy was measured among adults by the following question: “Thinking about your total monthly income, how difficult or easy is it for you to make ends meet?” Among children and adolescents (aged 10-17 y), perceived income adequacy was by asking, “Does your family have enough money to pay for things your family needs?”

We found a high level of correspondence between responses from children and adolescents and their parents or guardians. For example, of all parents or guardians who reported the family’s income adequacy as very difficult, 76% of children and adolescents reported their families as having barely enough or not enough money (Figure 3). Pearson correlation coefficients indicated moderate positive correlations between children and adolescents and their parents or guardians in reported income adequacy in Australia (*r* = 0.56), Canada (*r* = 0.59), Chile (*r* = 0.47), Mexico (*r* = 0.48), the United Kingdom (*r* = 0.61), and the United States (*r* = 0.62; all *P* < .001).

## Discussion

The current study is among the largest to compare alternative measures of financial position that are routinely collected in population-level survey research. Our findings add to the literature suggesting that income adequacy may be preferrable to traditional measures of household income for capturing financial well-being in the context of health.

In this large multicountry dataset, we found a lower proportion of missing data for perceived income adequacy than for household income. This difference is likely due to a greater willingness to divulge information on perceived income adequacy than on household income.^8^ Respondents may also find it more difficult to report household income, which requires quantitative estimates during a 12-month period and proxy reporting for an entire household. These estimates can be particularly difficult for respondents in households with regularly fluctuating income, such as those that rely on hourly or seasonal employment. Similarly, household income may be difficult to report for children, adolescents, and young adults, who often live in shared households, as well as for older adults, who have atypical income sources during retirement.^6,8^

Perceived income adequacy was positively correlated with household income, as expected. However, the correlations were weak to moderate, indicating a qualitative difference between the measures. As shown in previous literature, all levels of perceived income adequacy appeared across all household income groups; for example, some respondents in the lowest household income group reported high income adequacy.^5^ These discrepancies likely reflect the psychological nature and relativity of the income adequacy measure (eg, differences in respondents’ expectations or satisfaction with income sufficiency), as well as objective differences in nonincome factors (eg, cost of living, debt, access to other financial resources). These findings reinforce a potential limitation of perceived income adequacy in this respect: such measures are inherently constrained by the fact that perceived income adequacy can vary drastically between respondents of the same objective income level. Thus, it remains important to distinguish between perceived income adequacy and objective measures of income, which can have distinct meanings and affect health outcomes in different ways.^6,7^

The apparent differences observed between the measures may further explain why perceived income adequacy was a stronger predictor of household food security than household income. While neither perceived income adequacy nor household income was a particularly strong predictor—explaining, at most, 32% of the variance in household food security—perceived income adequacy came out as the stronger predictor in all countries, sometimes substantially. For example, in Australia, perceived income adequacy explained 24% of the variance in household food security as compared with only 6% explained by household income. In contrast, the 2 income measures appeared more closely matched in their ability to predict the food security of respondents in Mexico. These differences may be due in part to the greater proportion of respondents in Mexico with low education levels relative to the other countries. Overall, however, the results are consistent with previous research among people aged ≥15 years in Italy and among older adults in Britain, in which income adequacy was a better predictor of self-rated health than objective measures of income.^6,14^ The results also reflect existing evidence for other subjective measures of financial position, such as subjective social status and financial hardship, both of which have been shown to predict physical health outcomes beyond traditional objective indicators of socioeconomic status.^12,24-26^

When we examined these relationships across age, ethnicity, education, and employment groups, income adequacy remained a stronger predictor than household income in all subgroups and particularly among older adults (aged ≥60 y), retired respondents, and those reporting parental leave or long-term illness or disability. Notably, both income measures were stronger predictors of food security for respondents reporting a majority ethnicity than a minority ethnicity. These results reflect previous research suggesting that the relationship between subjective social status and physical health outcomes may be stronger among White respondents than among respondents in racial and ethnic minority groups.^24,27^

Lastly, this study found strong correspondence in income adequacy between children and adolescents and their parents or guardians. This correspondence represents a potentially important finding for assessing family socioeconomic status in surveys conducted among children and young people, which often rely upon children reporting parental education level, spending money, or other measures with known limitations.^9^

## Practice Implications

Overall, our findings suggest that perceived income adequacy is a suitable yet distinct alternative to traditional measures of household income. The measure may be particularly favorable for assessing the effect of financial position on health among young people and among older or retired populations for whom household income may be difficult to report. For public health researchers using surveys to explore financial well-being or socioeconomic status, a measure of income adequacy (in comparison with a traditional measure of household income) may help to reduce the cognitive effort required for participants, minimize data loss through nonresponse, and produce wholistic estimates of respondents’ financial well-being. Given that this dataset used listwise deletion to remove missing data from the analytic dataset, future research should explore how perceived income adequacy relates to household income and household food security status in data with multiple imputations. Additional research directly comparing the usefulness of other subjective measures, such as subjective social status, would help to provide clarity to researchers and practitioners on which tools might best elucidate the relationship between financial position and poor health.

## Supplemental Material

sj-docx-1-phr-10.1177_00333549251358655 – Supplemental material for Perceived Income Adequacy Versus Household Income as a Measure of Socioeconomic Status in 6 Countries, 2022-2023 International Food Policy Study Supplemental material, sj-docx-1-phr-10.1177_00333549251358655 for Perceived Income Adequacy Versus Household Income as a Measure of Socioeconomic Status in 6 Countries, 2022-2023 International Food Policy Study by Rachel B. Acton, Christine M. White, Vicki L. Rynard and David Hammond in Public Health Reports®
