# Supplementary material for: Perceived Income Adequacy Versus Household Income as a Measure of Socioeconomic Status in 6 Countries, 2022-2023 International Food Policy Study
Source: Public Health Rep. 2025 Aug 20;140(5-6):468–76. doi: 10.1177/00333549251358655 (PMC12367702; doi:10.1177/00333549251358655)
Supplement: sj-docx-1-phr-10.1177_00333549251358655 – Supplemental material for Perceived Income Adequacy Versus Household Income as a Measure of Socioeconomic Status in 6 Countries, 2022-2023 International Food Policy Study [file sj-docx-1-phr-10.1177_00333549251358655.docx]

**SUPPLEMENTAL MATERIAL**

**Methods for adjusting household income for household size**

Household income was queried among adult respondents in each country using the following measures:

| **Australia, Canada, United States** | **United Kingdom** | **Mexico** |
| --- | --- | --- |
| What was your total household income, from all sources, before taxes, over the past 12 months? Include income from work, investments, pensions or government. Include income from all family members living with you all, or most of the time. Do NOT include roommates unless you share income.  Less than $10,000  $10,000 to less than $20,000  $20,000 to less than $30,000  $30,000 to less than $40,000  $40,000 to less than $50,000  $50,000 to less than $60,000  $60,000 to less than $70,000  $70,000 to less than $80,000  $80,000 to less than $90,000  $90,000 to less than $100,000  $100,000 to less than $150,000  $150,000 and over  Don’t know  Refuse to answer | What was your total household income, from all sources, before taxes, over the past 12 months? Include income from work, investments, pensions or government. Include income from all family members living with you all, or most of the time. Do NOT include housemates unless you share income.  Less than £5,000  £5,000 to less than 10,000  £10,000 to less than £15,000  £15,000 to less than £20,000  £20,000 to less than £25,000  £25,000 to less than £30,000  £30,000 to less than £35,000  £35,000 to less than £40,000  £40,000 to less than £45,000  £45,000 to less than £50,000  £50,000 to less than £75,000  £75,000 to less than £100,000  £100,000 and over  Don’t know  Refuse to answer | What was your total household income, from all sources, before taxes, over the past MONTH? Include income from work, investments, pensions or government. Include income from all family members living with you all, or most of the time. Do NOT include roommates unless you share income.  Less than $3,000  $3,000 to less than $5,000  $5,000 to less than $7,000  $7,000 to less than 9,000  $9,000 to less than $11,000  $11,000 to less than $13,000  $13,000 to less than $16,000  $16,000 to less than $20,000  $20,000 to less than $26,000  $26,000 to less than $56,000  $56,000 and over  Don’t know  Refuse to answer |

Responses to the above measures were then adjusted for household size as follows:

1. Each income category was recoded as the mean income value for that category (e.g., *‘£40,000 to less than £45,000’* was recoded as *‘£42,500’*) or in the case of the minimum and maximum categories, a value higher or lower equivalent to the distribution of the other income category means (e.g., *‘Less than $10,000’* was recoded as *‘$5,000’*), as shown in the table below. 'Don't know' and 'Refuse to answer' were coded as missing.

| **Australia, Canada, United States** | **United Kingdom** | **Mexico** |
| --- | --- | --- |
| $5,000 $15,000 $25,000 $35,000 $45,000 $55,000 $65,000 $75,000 $85,000  $95,000 $125,000 $175,000 | £2,500 £7,500 £12,500 £17,500 £22,500 £27,500 £32,500 £37,500 £42,500 £47,500 £52,500 £77,500 £112,500 | $2,000 $4,000 $6,000 $8,000 $10,000 $12,000 $14,000 $18,000 $23,000 $41,000 $71,000 |

1. These recoded income values were then adjusted for household size for each respondent by dividing the values by the square root of the number of people living in the respondent’s household (self-reported elsewhere in the survey).*
2. The adjusted household income values were then categorized into quintiles for each country to allow for cross-country comparisons.

*This adjustment “addresses the fact that individuals living together can share resources and the marginal increase in need decreases as the number of individuals sharing resources increases”.^1^

**Reference:**

1. Statistics Canada. Adjusted after-tax income of economic family. 2021. Available from: <https://www23.statcan.gc.ca/imdb/p3Var.pl?Function=DEC&Id=103386>

**eTable S1.** Sociodemographic characteristics, perceived income adequacy, adjusted household income and household food security status of adult respondents in the 2022-2023 International Food Policy Study surveys, by country, weighted by survey weight

|  | **Australia** | | **Canada** | | **Mexico** | | **United Kingdom** | | **United States** | |
| --- | --- | --- | --- | --- | --- | --- | --- | --- | --- | --- |
|  | (N=8,217) | | (N=8,965) | | (N=11,372) | | (N=8,132) | | (N=14,227) | |
|  | % | (n) | % | (n) | % | (n) | % | (n) | % | (n) |
| **Age** |  |  |  |  |  |  |  |  |  |  |
| 18-29 years | 20.3 | (1666) | 19.5 | (1747) | 28.3 | (3214) | 18.6 | (1516) | 20.4 | (2907) |
| 30-44 years | 27.2 | (2237) | 25.8 | (2312) | 30.8 | (3502) | 25.2 | (2045) | 25.7 | (3658) |
| 45-59 years | 23.4 | (1921) | 23.2 | (2080) | 29.6 | (3361) | 25.2 | (2045) | 23.8 | (3388) |
| 60+ years | 29.1 | (2393) | 31.5 | (2826) | 11.4 | (1294) | 31.1 | (2526) | 30.0 | (4274) |
|  |  |  |  |  |  |  |  |  |  |  |
| **Sex** |  |  |  |  |  |  |  |  |  |  |
| Male | 49.1 | (4038) | 49.5 | (4437) | 48 | (5454) | 48.4 | (3934) | 49.1 | (6986) |
| Female | 50.9 | (4179) | 50.5 | (4528) | 52 | (5918) | 51.6 | (4198) | 50.9 | (7241) |
|  |  |  |  |  |  |  |  |  |  |  |
| **Gender** |  |  |  |  |  |  |  |  |  |  |
| Man | 48.7 | (4003) | 48.9 | (4380) | 47.5 | (5403) | 48.1 | (3909) | 48.4 | (6891) |
| Woman | 50.5 | (4153) | 49.8 | (4467) | 52.0 | (5914) | 51.4 | (4177) | 50.7 | (7209) |
| Trans male/trans man | 0.2 | (17) | 0.4 | (38) | 0.1 | (9) | 0.1 | (11) | 0.2 | (35) |
| Trans female/trans woman | 0.03 | (3) | 0.2 | (17) | 0.02 | (2) | 0.1 | (10) | 0.1 | (18) |
| Gender queer/gender non-conforming + different gender identity | 0.4 | (36) | 0.6 | (57) | 0.1 | (14) | 0.3 | (22) | 0.4 | (57) |
| Not stated / missing | 0.1 | (4) | 0.1 | (6) | 0.3 | (30) | 0.02 | (2) | 0.1 | (16) |
|  |  |  |  |  |  |  |  |  |  |  |
| **Education^a^** |  |  |  |  |  |  |  |  |  |  |
| Low | 37.5 | (3079) | 40.4 | (3626) | 76.3 | (8676) | 40.1 | (3259) | 55.0 | (7827) |
| Medium | 32.2 | (2646) | 31.5 | (2824) | 8.9 | (1015) | 25.1 | (2043) | 9.9 | (1412) |
| High | 29.9 | (2458) | 27.6 | (2478) | 14.7 | (1673) | 34.0 | (2762) | 34.7 | (4933) |
| Not stated / missing | 0.4 | (34) | 0.4 | (37) | 0.1 | (9) | 0.8 | (68) | 0.4 | (56) |
|  |  |  |  |  |  |  |  |  |  |  |
| **Ethnicity^b^** |  |  |  |  |  |  |  |  |  |  |
| Majority | 70.8 | (5814) | 74.8 | (6704) | 78.8 | (8961) | 84.8 | (6896) | 61.9 | (8802) |
| Minority | 29.0 | (2382) | 23.5 | (2110) | 18.7 | (2132) | 14.7 | (1196) | 37.8 | (5374) |
| Not stated | 0.3 | (21) | 1.7 | (150) | 2.5 | (280) | 0.5 | (41) | 0.4 | (51) |
|  |  |  |  |  |  |  |  |  |  |  |
| **Employment status^c^** |  |  |  |  |  |  |  |  |  |  |
| Paid work | 53.1 | (4364) | 47.8 | (4286) | 55.3 | (6289) | 50.4 | (4099) | 44.3 | (6306) |
| Unemployed or unpaid work | 22.4 | (1843) | 21.2 | (1904) | 37.2 | (4232) | 20.2 | (1641) | 28.0 | (3985) |
| Retired | 19.6 | (1614) | 24.4 | (2184) | 5.2 | (597) | 23.1 | (1877) | 21.6 | (3080) |
| Parental leave or long-term illness/disability | 3.7 | (304) | 4.6 | (412) | 0.8 | (96) | 5.4 | (436) | 3.2 | (455) |
| Other | 0.6 | (49) | 0.9 | (81) | 0.8 | (87) | 0.4 | (33) | 0.9 | (129) |
| Not stated | 0.5 | (42) | 1.1 | (98) | 0.6 | (72) | 0.6 | (46) | 1.9 | (273) |
|  |  |  |  |  |  |  |  |  |  |  |
| **Household income^d^** |  |  |  |  |  |  |  |  |  |  |
| Quintile 1 (lowest) | 17.1 | (1402) | 18 | (1610) | 18.4 | (2087) | 18.2 | (1478) | 18.4 | (2623) |
| Quintile 2 | 19.2 | (1574) | 19.3 | (1726) | 19.1 | (2176) | 19.1 | (1557) | 19.7 | (2801) |
| Quintile 3 | 19.6 | (1614) | 18.5 | (1660) | 19.5 | (2217) | 16.1 | (1309) | 18.7 | (2667) |
| Quintile 4 | 18.4 | (1514) | 18.9 | (1690) | 18.3 | (2076) | 20.5 | (1667) | 18.3 | (2601) |
| Quintile 5 (highest) | 19.7 | (1619) | 19 | (1702) | 19.9 | (2268) | 19.8 | (1607) | 20.9 | (2980) |
| Not stated / missing | 6 | (494) | 6.4 | (577) | 4.8 | (548) | 6.3 | (514) | 3.9 | (555) |
|  |  |  |  |  |  |  |  |  |  |  |
| **Perceived income adequacy^e^** |  |  |  |  |  |  |  |  |  |  |
| Very difficult | 8.5 | (698) | 10.7 | (959) | 15.9 | (1806) | 7.7 | (629) | 11.4 | (1617) |
| Difficult | 21.8 | (1795) | 21.2 | (1902) | 35.5 | (4042) | 22 | (1792) | 20.8 | (2957) |
| Neither easy nor difficult | 35.9 | (2952) | 36.1 | (3240) | 37.7 | (4285) | 37.5 | (3050) | 31.9 | (4542) |
| Easy | 24.3 | (1995) | 21 | (1886) | 8.2 | (928) | 21.9 | (1784) | 20.2 | (2867) |
| Very easy | 8.4 | (694) | 9.8 | (877) | 2.1 | (238) | 9.8 | (795) | 14.7 | (2097) |
| Not stated / missing | 1 | (83) | 1.1 | (102) | 0.7 | (74) | 1 | (83) | 1 | (146) |
|  |  |  |  |  |  |  |  |  |  |  |
| **Household food security status^f^** |  |  |  |  |  |  |  |  |  |  |
| Food secure | 61.8 | (5077) | 62.6 | (5615) | 37.6 | (4271) | 64 | (5202) | 54.7 | (7780) |
| Low food security | 16.4 | (1349) | 16.7 | (1498) | 28.7 | (3265) | 14.8 | (1201) | 16.8 | (2387) |
| Very low food security | 21.3 | (1754) | 19.9 | (1788) | 33.5 | (3815) | 20.6 | (1679) | 27.7 | (3945) |
| Not stated / missing | 0.5 | (37) | 0.7 | (64) | 0.2 | (21) | 0.6 | (50) | 0.8 | (115) |
| ^a^ Participants were asked, “What is the highest level of formal education that you have completed?” Responses were categorized as ‘low’ (completed secondary school or less), ‘medium’ (some post-secondary qualifications), or ‘high’ (university degree or higher) according to country-specific criteria. | | | | | | | | | | |
| ^b^ Ethnicity was assessed using country‑specific race/ethnicity categories and was analyzed as a binary variable (majority/minority) to accommodate comparisons across countries. Ethnicity categories were recoded as follows: (1) Australia majority = only speaks English at home, minority = speaks a language other than English at home or indicated they are aboriginal or Torres Straight Islander; (2) Canada majority = White, minority = other ethnicity; (3) Mexico majority = non‑Indigenous, minority = Indigenous; (4) UK majority = White, minority = other ethnicity; (5) US majority = White, minority = other ethnicity. | | | | | | | | | | |
| ^c^ Participants were asked, “What was your main activity in the past week?” Responses were categorized into ‘paid work’ (working at a paid job or business; vacation (from paid work)), ‘unemployed or unpaid work’ (looking for paid work; going to school (including vacation from school); caring for children; household work; volunteering; caregiving other than for children), ‘retired’ (retired), ‘parental leave or long-term disability’ (maternity/paternity leave; long term illness (or disability)), or ‘other’ (other open-text responses – e.g., short term illness). | | | | | | | | | | |
| ^d^ Adjusted household income was assessed by asking respondents to select their total household income from a range of 11-13 response options, depending on the country. Reported household income was then adjusted for self-reported household size and categorized into quintiles. | | | | | | | | | | |
| ^e^ Perceived income adequacy was assessed by asking, “Thinking about your total monthly income, how difficult or easy is it for you to make ends meet?” | | | | | | | | | | |
| ^f^ Household food security was assessed using the 18-item Household Food Security Survey Module. | | | | | | | | | | |

**eTable S2.** Sociodemographic characteristics and perceived income adequacy of youth respondents and their parent/guardian panelists in the 2022-2023 International Food Policy Study surveys, by country, weighted by survey weight

|  | **Australia** | | **Canada** | | **Chile** | | **Mexico** | | **United Kingdom** | | **United States** | |
| --- | --- | --- | --- | --- | --- | --- | --- | --- | --- | --- | --- | --- |
|  | (N=2,667) | | (N=7,422) | | (N=3,151) | | (N=3,189) | | (N=3,202) | | (N=3,382) | |
|  | % | (n) | % | (n) | % | (n) | % | (n) | % | (n) | % | (n) |
| **Age** |  |  |  |  |  |  |  |  |  |  |  |  |
| 10-13 years | 50.8 | (1354) | 50.2 | (3723) | 48.4 | (1525) | 50.4 | (1608) | 51.3 | (1641) | 49.0 | (1658) |
| 14-17 years | 49.2 | (1313) | 49.8 | (3699) | 51.6 | (1626) | 49.6 | (1581) | 48.7 | (1561) | 51.0 | (1724) |
|  |  |  |  |  |  |  |  |  |  |  |  |  |
| **Sex** |  |  |  |  |  |  |  |  |  |  |  |  |
| Male | 51.4 | (1372) | 51.1 | (3793) | 51.1 | (1610) | 50.7 | (1618) | 51.3 | (1642) | 51.2 | (1732) |
| Female | 48.6 | (1295) | 48.9 | (3629) | 48.9 | (1541) | 49.3 | (1571) | 48.7 | (1560) | 48.8 | (1650) |
|  |  |  |  |  |  |  |  |  |  |  |  |  |
| **Ethnicity^a^** |  |  |  |  |  |  |  |  |  |  |  |  |
| Majority | 68.4 | (1823) | 65.8 | (4881) | 83.2 | (2621) | 77.1 | (2459) | 75.4 | (2415) | 49.9 | (1688) |
| Minority | 31.3 | (835) | 32.7 | (2429) | 14.4 | (454) | 19.3 | (616) | 23.8 | (762) | 49.6 | (1677) |
| Not stated | 0.3 | (8) | 1.5 | (112) | 2.4 | (76) | 3.6 | (113) | 0.8 | (25) | 0.5 | (17) |
|  |  |  |  |  |  |  |  |  |  |  |  |  |
| **Youth perceived income adequacy^b^** |  |  |  |  |  |  |  |  |  |  |  |  |
| Not enough money | 5.3 | (140) | 4.4 | (329) | 6.3 | (199) | 4.5 | (142) | 4.8 | (153) | 6.6 | (225) |
| Barely enough money | 19.9 | (531) | 21.0 | (1560) | 32.6 | (1026) | 30.1 | (959) | 22.9 | (733) | 24.2 | (819) |
| Enough money | 59.3 | (1581) | 59.8 | (4442) | 56.1 | (1767) | 58.7 | (1871) | 60.5 | (1939) | 52.2 | (1766) |
| More than enough money | 14.2 | (379) | 13.1 | (971) | 3.3 | (105) | 5.8 | (185) | 9.7 | (311) | 15.6 | (529) |
| Not stated / missing | 1.4 | (36) | 1.6 | (120) | 1.7 | (53) | 1.0 | (32) | 2.1 | (67) | 1.3 | (43) |
|  |  |  |  |  |  |  |  |  |  |  |  |  |
| **Parent/guardian perceived income adequacy^c^** |  |  |  |  |  |  |  |  |  |  |  |  |
| Very difficult | 11.0 | (293) | 10.2 | (758) | 17.1 | (539) | 9.3 | (298) | 8.3 | (265) | 14.2 | (481) |
| Difficult | 27.2 | (725) | 29.4 | (2185) | 38.8 | (1223) | 30.5 | (971) | 31.2 | (998) | 27.5 | (930) |
| Neither easy nor difficult | 32.3 | (861) | 34.7 | (2574) | 34.4 | (1085) | 37.0 | (1180) | 36.3 | (1162) | 26.2 | (886) |
| Easy | 20.9 | (558) | 18.0 | (1337) | 7.1 | (225) | 17.1 | (546) | 17.8 | (569) | 18.6 | (628) |
| Very easy | 8.3 | (222) | 7.0 | (518) | 1.5 | (49) | 5.7 | (182) | 6.2 | (198) | 13.1 | (444) |
| Not stated / missing | 0.3 | (8) | 0.7 | (50) | 1.0 | (30) | 0.4 | (12) | 0.3 | (10) | 0.4 | (12) |
| ^a^ Ethnicity was assessed using country‑specific race/ethnicity categories and was analyzed as a binary variable (majority/minority) to accommodate comparisons across countries. Ethnicity categories were recoded as follows: (1) Australia majority = only speaks English at home, minority = speaks a language other than English at home or indicated they are aboriginal or Torres Straight Islander; (2) Canada majority = White, minority = other ethnicity; (3) Chile majority = non Indigenous, minority = Indigenous; (4) Mexico majority = non‑Indigenous, minority = Indigenous; (5) UK majority = White, minority = other ethnicity; (6) US majority = White, minority = other ethnicity. | | | | | | | | | | | | |
| ^b^ Youth perceived income adequacy was assessed by asking youth, “Does your family have enough money to pay for things your family needs?” | | | | | | | | | | | | |
| ^c^ Parent/guardian perceived income adequacy was assessed by asking the youth respondent’s parent/guardian, “Thinking about your total monthly income, how difficult or easy is it for you to make ends meet?” | | | | | | | | | | | | |

**eTable S3.** Percent missing responses for adjusted household income and perceived income adequacy among adults in the 2022-2023 International Food Policy Study surveys, across all countries (N=50,913), overall and by age, education and perceived income adequacy groups, weighted by survey weight

|  | **Missing adjusted household income^a^** | | **Missing perceived income adequacy^b^** | |
| --- | --- | --- | --- | --- |
|  | % | (n) | % | (n) |
| **Overall** | 5.3 | (2688) | 1.0 | (488) |
|  |  |  |  |  |
| **Age** |  |  |  |  |
| 18-29 years | 7.1 | (788) | 2.4 | (262) |
| 30-44 years | 3.8 | (525) | 0.7 | (90) |
| 45-59 years | 5.0 | (643) | 0.6 | (82) |
| 60+ years | 5.5 | (736) | 0.4 | (53) |
|  |  |  |  |  |
| **Education^c^** |  |  |  |  |
| Low | 5.7 | (1502) | 1.1 | (289) |
| Medium | 5.1 | (507) | 0.7 | (70) |
| High | 4.1 | (593) | 0.4 | (63) |
| Not stated / missing | 43.0 | (88) | 32.5 | (66) |
|  |  |  |  |  |
| **Perceived income adequacy^b^** |  |  |  |  |
| Very difficult | 4.8 | (276) | - | - |
| Difficult | 4.4 | (548) | - | - |
| Neither easy nor difficult | 5.8 | (1049) | - | - |
| Easy | 3.9 | (374) | - | - |
| Very easy | 3.6 | (169) | - | - |
| Not stated / missing | 55.8 | (272) | - | - |
| ^a^ Adjusted household income was assessed by asking respondents to select their total household income from a range of 11-13 response options, depending on the country. Reported household income was then adjusted for self-reported household size and categorized into quintiles. | | | | |
| ^b^ Perceived income adequacy was assessed by asking, “Thinking about your total monthly income, how difficult or easy is it for you to make ends meet?” | | | | |
| ^c^ Participants were asked, “What is the highest level of formal education that you have completed?” Responses were categorized as ‘low’ (completed secondary school or less), ‘medium’ (some post-secondary qualifications), or ‘high’ (university degree or higher) according to country-specific criteria. | | | | |
